# Orthodontic Protocol Using Mini-Implant for Class II Treatment in Patient with Special Needs

**DOI:** 10.1155/2016/1057263

**Published:** 2016-10-26

**Authors:** Fernando Pedrin Carvalho Ferreira, Anderson Paulo Barbosa Lima, Eliana de Cássia Molina de Paula, Ana Claudia de Castro Ferreira Conti, Danilo Pinelli Valarelli, Renata Rodrigues de Almeida-Pedrin

**Affiliations:** ^1^CORA Vilhena, Vilhena, RO, Brazil; ^2^Department of Orthodontics, Universidade do Sagrado Coração, Bauru, SP, Brazil

## Abstract

Improving facial and dental appearance and social interaction are the main factors for special needs (SN) patients to seek orthodontic treatment. The cooperation of SN patients and their parents is crucial for treatment success.* Objective*. To show through a case report the satisfactory results, both functional and esthetic, in patients with intellectual disability, congenital nystagmus, and severe scoliosis.* Materials Used*. Pendulum device with mini-implants as anchorage unit.* Results*. Improvement of facial and dental esthetics, correction of Class II malocclusion, and no root resorption shown in the radiographic follow-up.* Conclusion*. Knowing the limitations of SN patients, having a trained team, motivating and counting on the cooperation of parents and patients, and employing quick and low-cost orthodontic therapy have been shown to be the essential factors for treatment success.

## 1. Introduction

Special needs (SN) patients are the ones who do not engage in normal activities of their age groups. The prevalence of severe malocclusions in these patients is high, which requires orthodontic therapy [1]. The aim of the orthodontic treatment is not only functional but also esthetic improvement [2]. Individuals with SN face a social acceptance barrier, while the improvement of dental esthetics has a positive influence on social interaction, increasing the chances of employment toward self-sufficiency. However, the effectiveness of the orthodontic treatment is limited and not satisfactory for all cases [3].

The concern with facial appearance is what triggers parents to seek orthodontic treatment. Although parents are motivated by the improvement of quality of life of their children, SN patients are less likely to receive treatment. Usually, few SN patients end up being treated, and the main reasons are the fear of parents regarding the cooperation of their children during treatment and finding a trained dental team [4]. The cooperation of SN patients and their parents is essential for the success of orthodontic treatment [1, 4]. The progress over the last years of medical science allowed great developments in the treatment of SN patients in both expectancy and quality of life. The demand for dental treatment from patients with some type of systemic disease and physical or mental disability and elderly and immunocompromised patients is increasingly high. Acknowledging the pathology and its implications for dental treatment is critical for the success of the therapy applied [5].

Patients with Class II malocclusion are the ones who mostly seek orthodontic treatment [6]. This malocclusion is characterized by maxillary prognathism, mandibular retrognathism, or the association of both [7, 8]. There are several therapeutic possibilities for treatment such as upper molar distalization, which may be performed with extraoral and intraoral appliances [9, 10]. In the case of SN patients, their lack of cooperation during treatment is a determinant factor for selecting the therapy to be applied. The use of intraoral distalizers is an alternative to be considered in these cases. Currently, there is a large diversity of intraoral devices in the market, and the pendulum device stands out among them because of the ease of clinical handling and its efficiency in correcting Class II malocclusion [11].

With the appearance of the mini-implant as temporary skeletal anchorage with easy insertion and removal in many areas of the maxilla and mandible, a new outlook was imposed regarding intraoral distalization [9, 12]. Associating the mini-implant as skeletal anchorage for the pendulum device would cancel its negative effects, which has already been described in the literature, improving treatment efficiency and reducing its time.

According to the above, this work aimed to show through a case report the satisfactory results, both functional and esthetic, in patients with intellectual disability, congenital nystagmus, and severe scoliosis. It also aimed to stress the importance of parental and patient cooperation and a trained dental team for treatment success.

## 2. Case Report

We report the case of a female patient, 1.55 m, 49 kg, 25 years old, with suggestive medical history for congenital nystagmus, severe scoliosis, and visual disability. Further information about the patient, such as past medical history, allergies, medication, and social and family history, is shown in Table 1.

**Figure 1:**
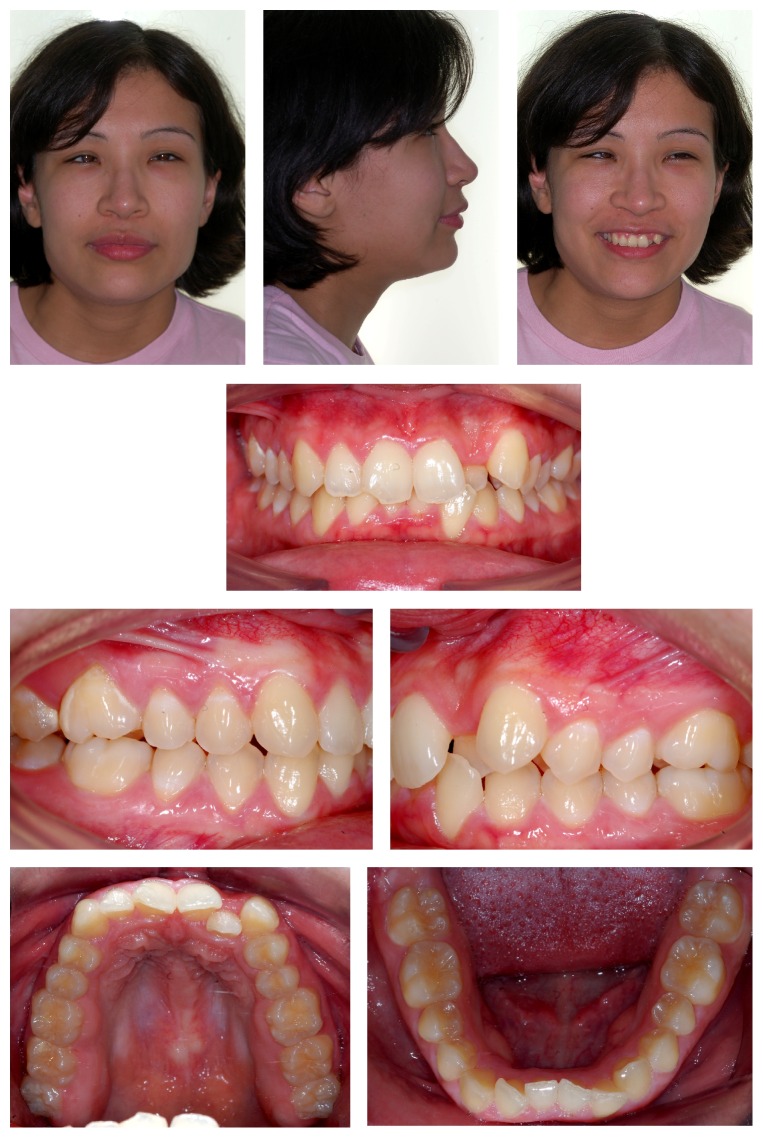
Extraoral and intraoral initial photos.

Treatment started with passive lip seal, acceptable facial asymmetry, and closed nasolabial angle. There was prevalence of horizontal growth (Figure 1). The intraoral evaluation revealed the presence of severe crowding in both upper and lower arches, upper canine in buccal-version, and left upper lateral incisor palatal tipped in corssbite position. There are half cusp (1/2) class II malocclusion on the right side and 3/4 cusp class II on the left side and overjet of 2 mm and overbite of 5 mm (Figure 1).

**Figure 2:**
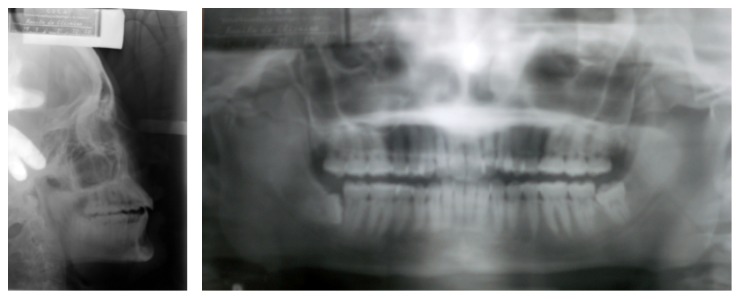
Initial panoramic radiography and teleradiography.

After assessing the panoramic radiography and teleradiography (Figure 2), the patient was diagnosed with maxillary retrusion, thus causing natural compensatory proclination of upper incisors. Mandible was normal with lower incisors well positioned in the symphysis (Table 1).

The options given to the parents of patients were exodontia of third upper molars and first upper premolars (teeth 14 and 24) even with the prevalence of horizontal growth, considering that the amount of crowding would fill the extraction space and the retraction of the anterior quadrant would be small, thus not aggravating the overbite. However, parents refused this option because of the high number of extractions and the potential lack of patient cooperation during surgical therapy. The second option, approved by the parents, was distalization with pendulum supported in mini-implant as skeletal anchorage. As such, the main objectives of the treatment would be achieved: distalization of first upper molars toward Class I position and improvement of dental and facial esthetics.

**Figure 3:**
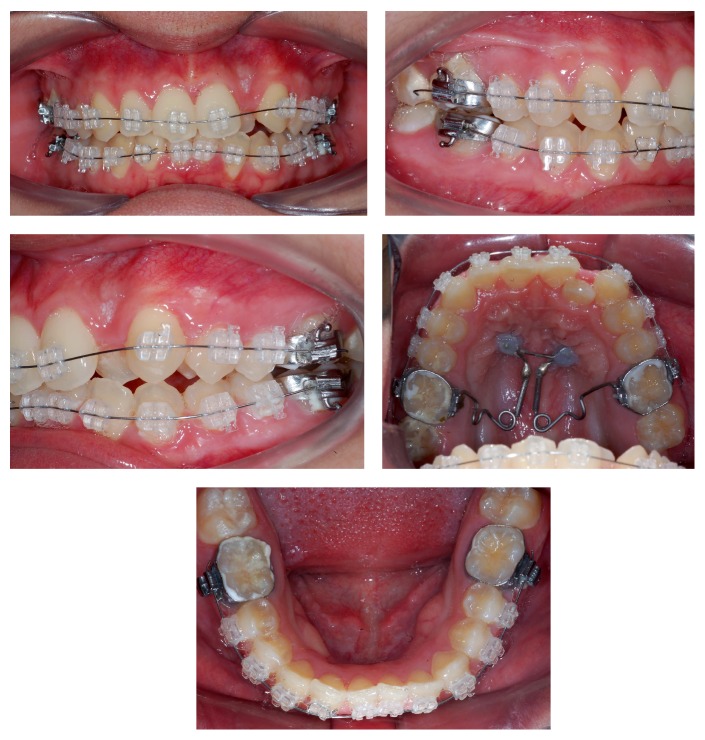
Bonding of upper and lower fixed appliances after one month from the start of distalization with pendulum.

The treatment had a few steps. First, exodontia of teeth 18 and 28 was requested subsequently under local anesthesia (2% lidocaine hydrochloride with 1 : 50,000 norepinephrine hemitartrate); then, 2 titanium mini screws (SIN, São Paulo, Brazil) of 1.6 cm in diameter and 8 mm in length were installed in the hard palate area, not parallel to each other. After installation, an alginate molding was made and forwarded to the production of the pendulum's anchorage unit, where it would be bonded with photoresin (Figure 3).

**Figure 4:**
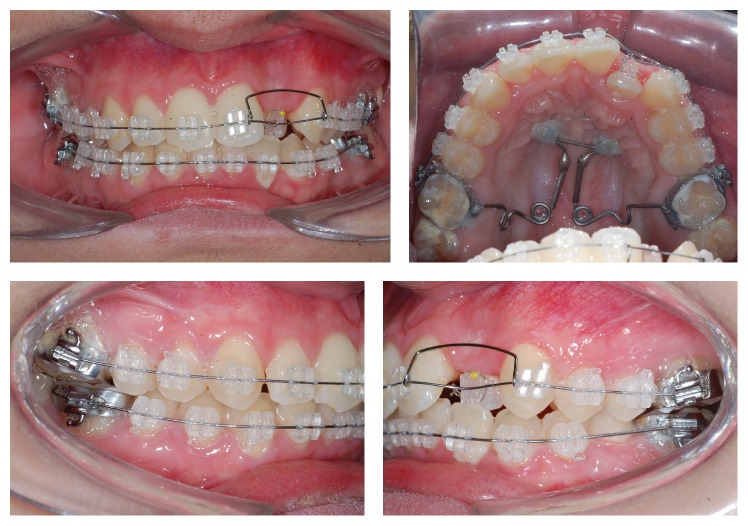
Start of tooth 22 alignment. JS spring used for space opening. Buccal torque of first upper molars.

**Figure 5:**
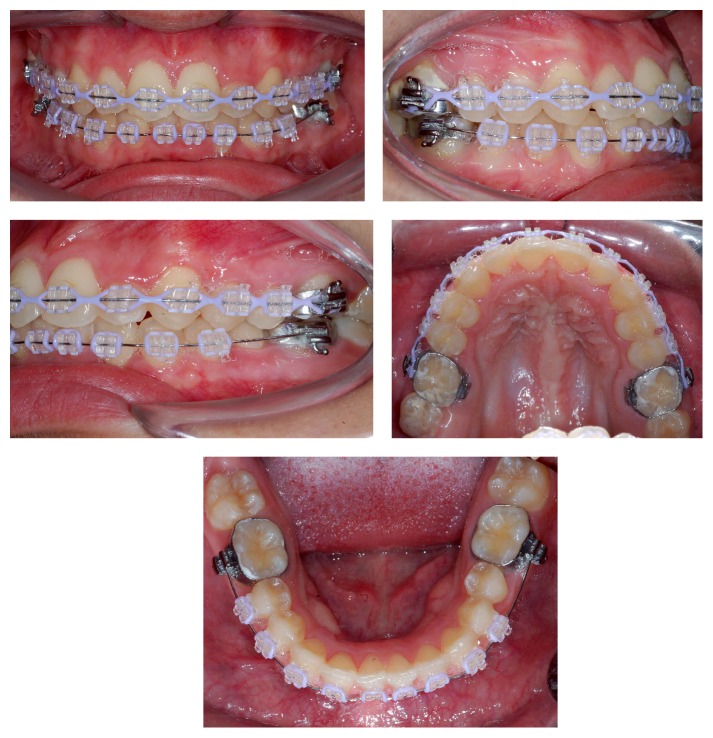
Class I upper molars and closing of remaining spaces with chain elastics.

After 1 month from the start of distalization of first upper molars, fixed orthodontic appliances were bonded to the upper and lower arches: Roth prescription (Iceram, Orthometric, Marília, SP, Brazil), 0.022′′ × 0.0028′′ slot with 0.014′′ Flexy Super Elastic, Orthometric, wire. A bracket was not bonded to tooth 22 for lack of space in the dental arch (Figure 4). In the fifth month of treatment, the first molars were in Class I, with accentuated buccal torque (Figure 4) and decreasing upper and lower crowding. In this step, the pendulum was removed along with the anchorage unit. Then, space opening for tooth 22 started, with spring (JS) produced with a 0.018′′ steel wire. In the tenth month of treatment, the remaining spaces were closed with chain elastics and with the installation of a 0.019′′ × 0.025′′ steel wire in the upper arch, improving the torque in the first upper molars (Figure 5); a 0.018′′ steel wire was installed in the lower arch. Folds (offset) were applied in the area of teeth 33 and 43, seeking the proper lateral movement (Figure 5).

**Figure 6:**
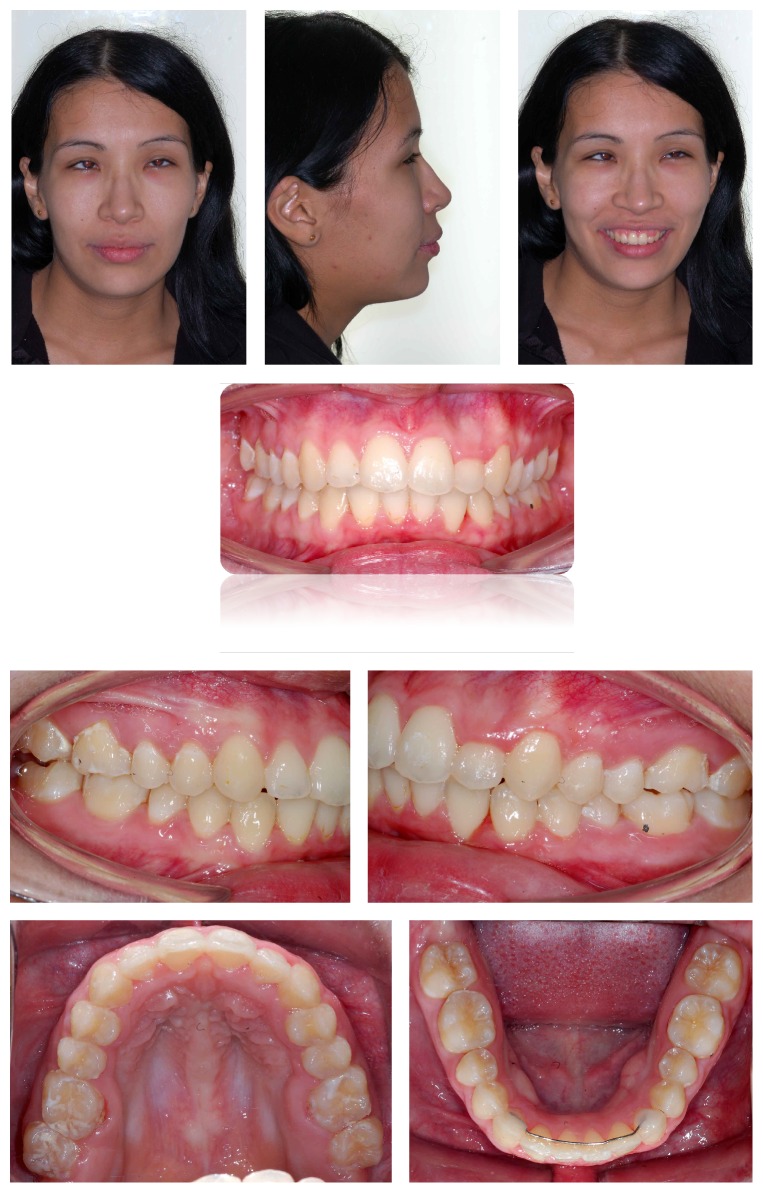
Removal of fixed appliance and bonding of 3 × 3 retainer on the lower arch 11 months after treatment started.

**Figure 7:**
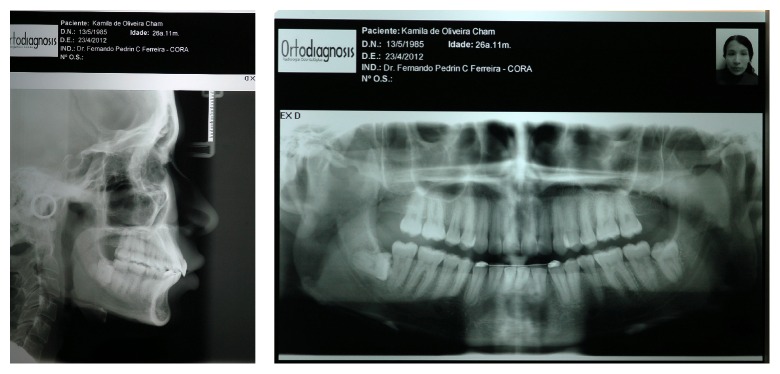
Final panoramic radiography and teleradiography.

After 11 months from the start of the treatment, the intercuspation procedure began. By the end of intercuspation and occlusal adjustment, the orthodontic fixed appliance was removed and retainers were produced. A Hawley plate was installed in the upper arch and a 3 × 3 fixed retainer was installed in the lower arch. Orthodontic therapy lasted 12 months (Figures 6 and 7).

**Figure 8:**
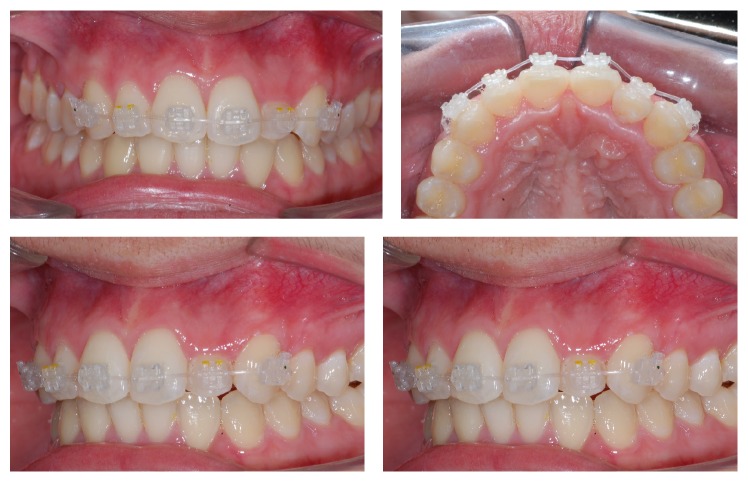
Rebonding of fixed appliance to correct relapse.

**Figure 9:**
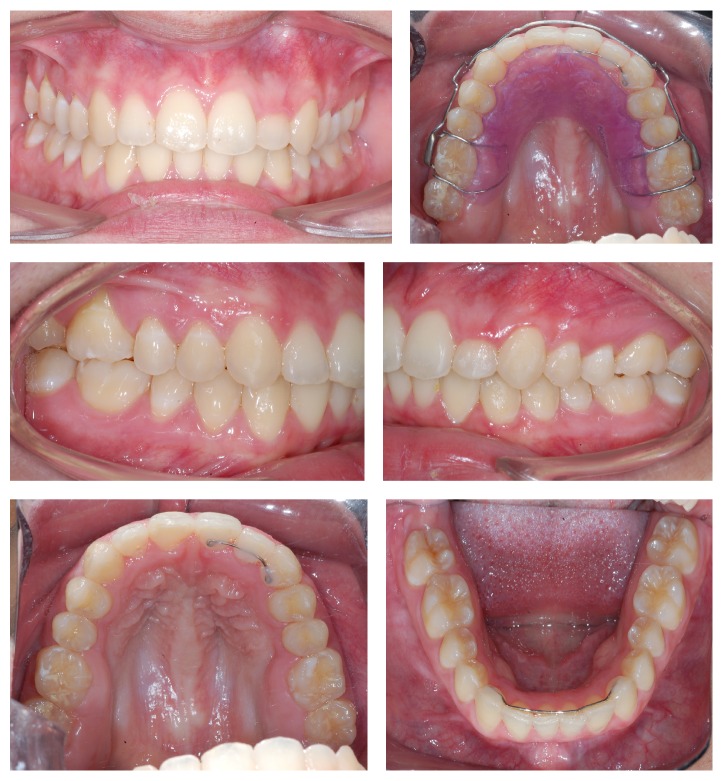
After 3 months, the fixed appliance was removed again and an upper fixed retainer was produced from tooth 21 to tooth 23.

In the first posttreatment control, one month after the removal of the fixed appliance, the upper anterior teeth were rebonded for the repositioning of tooth 22, which presented lingual relapse (Figure 8) due to the lack of patient cooperation in using the upper removable retainer. Releveling lasted 3 months, and after removal a fixed retainer was installed on teeth 21, 22, and 23 (Figure 9).

## 3. Results

**Figure 10:**
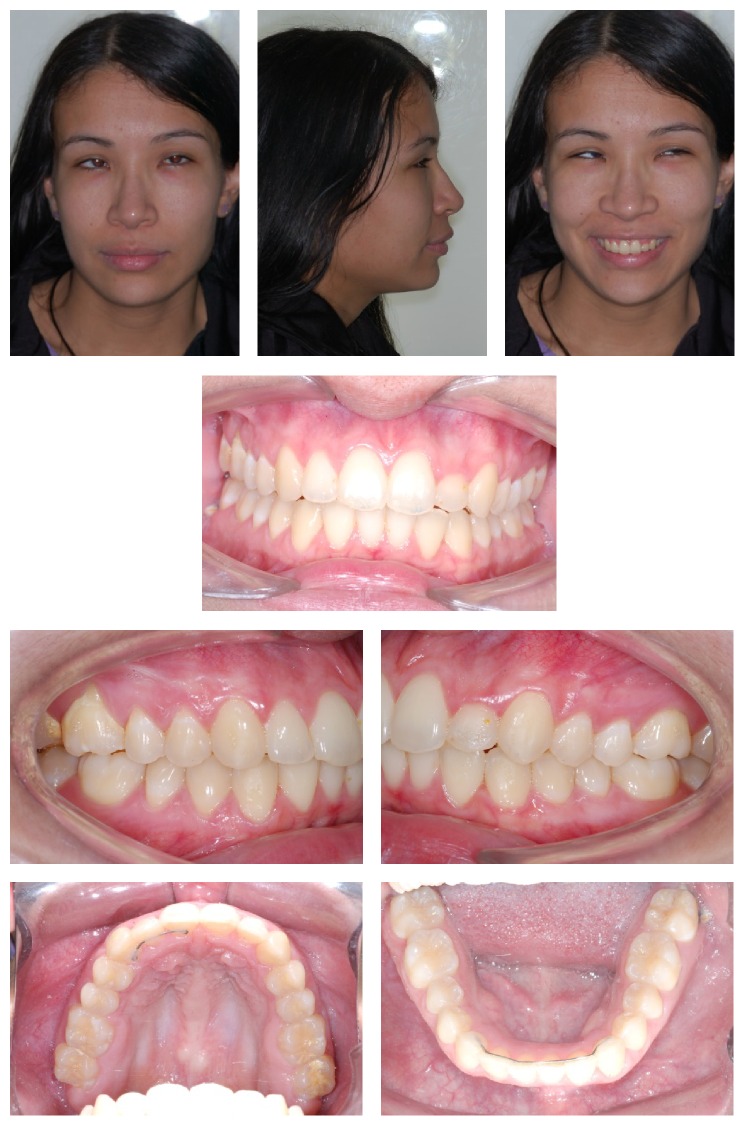
Extraoral and intraoral final photos showing improved facial and dental esthetics.

**Figure 11:**
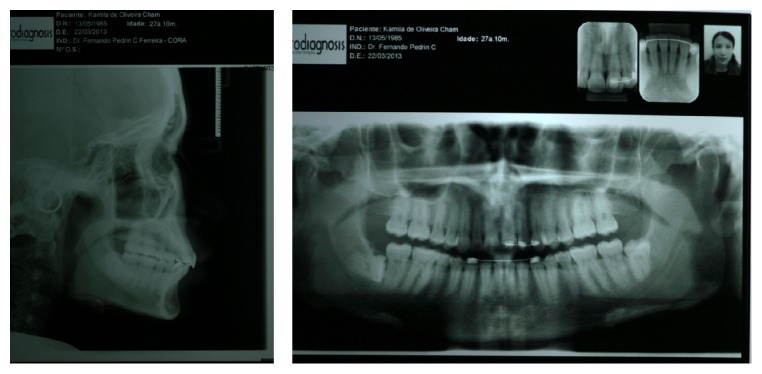
Final panoramic radiography and teleradiography. There was no significant root resorption.

**Figure 12:**
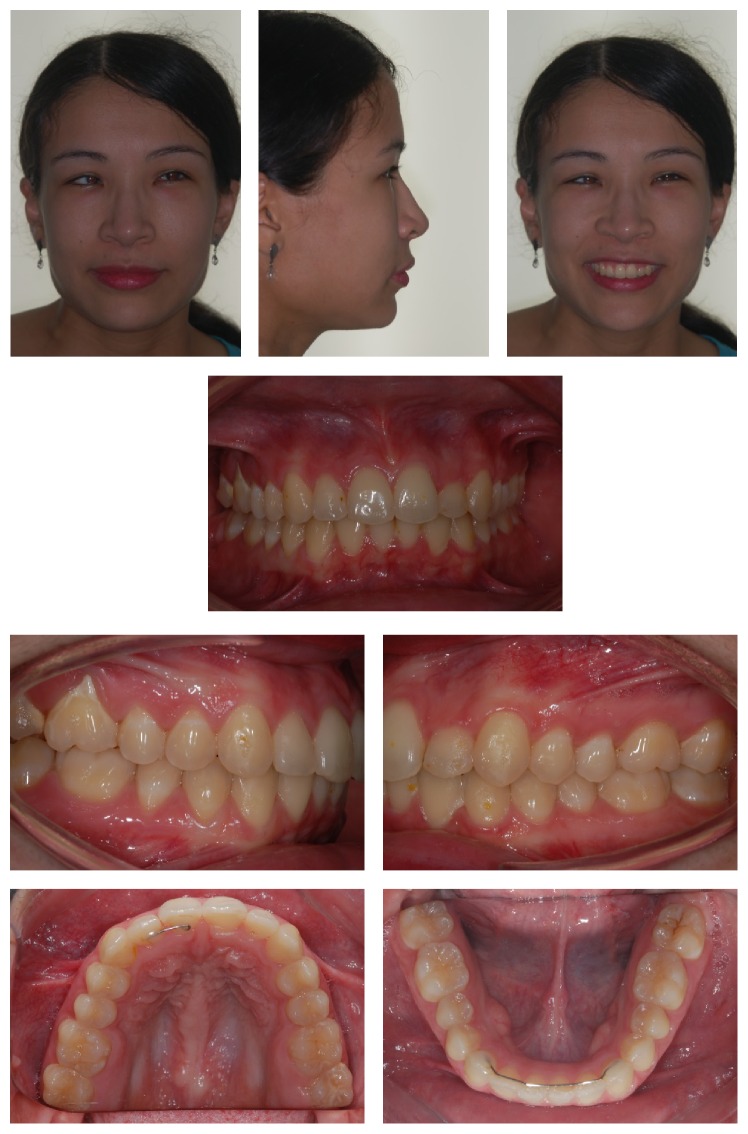
Extraoral and intraoral photos 34 months after the end of treatment.

There was an improvement in facial and dental esthetics and retrusion and verticalization of upper incisors, which improved profile and opening of the nasolabial angle, promoting facial balance and harmony (Figure 10). The dental relationship was obtained from an upper tooth with two lower teeth. Overjet and overbite were normal, and maxillary and mandibular median lines coincided. Radiographic follow-up showed no major resorption in teeth roots (Figure 11). Upper molars distalization occurred with the translation movement, which is considered ideal (Figure 11). After 34 months from the end of treatment, the patient was reassessed (Table 2). The results achieved were proven to be stable (Figure 12).

## 4. Discussion

In Dentistry, the term “special needs patient” includes not only children and adults that take medication for systemic diseases and people with motor impairments and intellectual disabilities but also patients with oral cavity disease, which makes dental treatment more complicated. Therefore, the term “special needs patient” includes every patient that requires a broad overview and a thorough physical, psychic, and social assessment in order to provide the correct treatment [5].

Waldman et al. [13] raised the question, “Do disabled people need esthetic and functional considerations to be comparable to ‘normal' people?” Improved physical appearance and oral function after orthodontic therapy could increase the quality of life of people with SN and promote better social acceptance [14]. Improved facial appearance and social integration are the major motivators for parents to seek orthodontic treatment [14]. Moreover, people with SN are more likely to present periodontal disease, causing severe esthetic malocclusions that hinder social relations and employment opportunities [13].

Children and adolescents with SN present higher prevalence of malocclusions than the normal population due to deleterious habits (thumb sucking, mouth breathing, and tongue interposition), different diet (no intake of solid food which requires thorough mastication), increased levels of caries, and early teeth loss. However, malocclusion may have evolved as postpartum trauma, prenatal effects, hereditary factors, or muscle development [13]. Class II malocclusion affects 33.7% of these patients [4].

The treatment for Class II malocclusion varies according to etiology, dentoalveolar involvement, and skeletal discrepancy. Several protocols are described in the literature, including dental extractions, functional orthopedic appliances, distalizers, or orthodontic/surgical treatment [6]. Because of the limitations of the patient reported in this study and the refusal from parents for a high number of extractions, the distalization of molars with pendulum supported in mini-implant was the more likely option to be performed. The mini-implant support eliminated the unwanted effects such as protrusion of anterior teeth (incisors and canines), mesial movement of premolars, and the increase of overjet, which would extend treatment time [6]. The study by Öncağ et al. [15] on the efficiency of the pendulum device supported in palate mini-implants concluded that there was no protrusion of upper incisors during the distalization process, reducing treatment time and presenting satisfactory esthetics and stable occlusion. The pendulum is a device that presents efficiency in upper molar distalization, eliminating the factor of patient cooperation; it is a low-cost device that is easy to produce and install [11, 16]. The use of extraoral appliances is the best option, but due to esthetic standards imposed by society, it is harder to get patient approval, which consequently leads to treatment failure [6]. The lack of cooperation from the patient exposed was another determinant factor for the selection of the pendulum device supported in mini-implants.

With the lack of cooperation of SN patients, parental participation is crucial for the success of orthodontic therapy [17]. Patient motivation does not increase over the different treatment steps, which is influenced by the presence of discomfort and the level of acceptance of the device employed. Parents are significantly more motivated than their children [18].

People with SN are used to receive constant daily attention from their motivated parents who are willing to do everything possible to improve the well-being of their children and are willing to become members of the team [14]. The dental team that receives SN patients and subsequently provides care should assess every aspect of the patient such as communication method, anxiety, and difficulties or challenges concerning behavior in order to maximize the potential for a positive result, which is important for the patient as to make it a successful experience. Some of the precautions to minimize anxiety are online media (websites and blogs) so that patients and parents have access to information about the practice they might experience, brochures showing the patient what might happen during their visit, and accessibility [19].

Usually, SN patients need to be sedated for dental procedures. Currently, propofol and midazolam are the primary agents used for sedation in dental treatment because of their short half-life and amnesic effects [20, 21]; the most common effect of these drugs is somnolence [22]. Special needs patients often take several drugs and the side effects may affect oral health. Anticonvulsants may cause gingival hyperplasia, and psychotropic and cardiovascular drugs may lead to xerostomia. The high level of sugar in medicines for children may contribute to dental caries [13]. Sedation was not required to install the mini-implants and other orthodontic appliances in the patient treated; only local anesthetic and previous topical anesthetic were needed. Parents were motivated and the patient was conditioned with the procedures to be performed.

Conventional intraoral distalizers take an average of 4–7 months to achieve molar Class I [23], although the literature imposes that the protocol of extraction of two upper premolars is faster than distalization to correct Class II [23]. The use of the pendulum device supported in mini-implants showed efficiency in correcting this malocclusion in a reduced time with satisfactory occlusal results and interesting biological cost, considering the limitations of the patient exposed.

One of the main objectives of the orthodontic treatment is to improve facial esthetics. Nose, lips, and chin should form a gentle outline of the face when seen in profile [24]. It is possible to observe slight retrusion and verticalization of upper incisors and a small opening of the nasolabial angle. Although the cephalometric result was not significant for retrusion and verticalization of upper incisors, the upper lip was posteriorly positioned, making the profile look straighter. Parents noticed the change. In the study by Bowman and Johnston Jr. [25], orthodontists and lay people had the same perception of the changes in profile after treatment. On the other hand, Cochrane et al. [26] affirm that dentists tend to be more critical than parents and patients regarding the perception of facial esthetics [26]. This corroborates with the study by Pithon et al. [27], where lay people assessed the profile of a female patient with accentuated bimaxillary protrusion. The image was altered to produce a series of photos with different lip positions, and, in conclusion, the straight profile was elected to be the most attractive. The study supports the understanding that people look beyond cephalometric measurements to assess facial appearance [25].

Gingival esthetics in the area of tooth 22 could be improved by finishing folds and arch twists (root buccal torque) [28], the root would be directed toward the buccal side, and root displacement followed by the alveolar bone would make the gingiva thinner, improving smile esthetics. However, parents refused such procedure, considering that the result presented exceeded their expectations. In a study performed in healthy adolescents who received orthodontic treatment, only 34% were completely satisfied with the results, 62% were relatively satisfied, and 4% were dissatisfied [29]. The study by Abeleira et al. [4] with disabled children resulted in 100% of satisfaction from the parents interviewed, and more than 40% affirmed that the results of the orthodontic treatment had exceeded their expectations.

Inadequate oral hygiene may be the greatest obstacle for orthodontic treatment success. People with SN may not understand the need for oral hygiene or present physical limitations [13]. The patient treated presented good oral hygiene, no dental caries, and low level of biofilm. The hygiene routine proposed by the parents was effective.

The “ghost” of dental treatment in SN patients is a label given by dental professionals. The lack of training of professionals and their teams may be the greatest aggravating factor. Knowing the limitations of these patients, providing a friendly environment, and motivating parents were the key factors for a satisfactory orthodontic treatment for both.

## 5. Conclusion

The treatment of a disabled patient requires special care. Patient motivation and conditioning added by parent cooperation are essential factors for treatment success. The selection of adequate orthodontic mechanics and the presence of a trained team are determinant factors for a more favorable prognosis.

## Figures and Tables

**Table 1:** Patient information.

Patient demographics	25-year-old Caucasian female 155 cm, 49 kg
Medical history	Nistagmus Scoliosis Visual disability
Allergies	Penicillin
Medications	Topiramate
Social history	Denies
Family history	Mother: none Father: none

**Table 2:** Cephalometric measurements.

Measurement	Initial (10/10)	Final (4/12)	Follow-up (3/13)
SNA	80.89	80.22	80.22
SNB	77.12	77.32	76.93
ANB	3.77	2.90	3.29
SN-MP	26.93	26.62	27.60
SN.Gn	70.12	70.23	70.50
FMA	17.13	17.84	16.87
1.1	128.17	112.55	119.48
1.NA	28.08	20.66	19.17
1-NA	7.40	6.70	6.27
1.NB	45.64	43.89	38.06
1-NB	7.78	7.63	7.31
IMPA	122.45	119.96	113.53
